# RND3 restricts encephalomyocarditis virus replication by promoting IKKε ubiquitination and type I interferon production

**DOI:** 10.1128/spectrum.01745-25

**Published:** 2025-12-15

**Authors:** Peng Ma, Zheng Wang, Zhengli Liu, Qi Xie, Tianxu Liu, Linjie Li

**Affiliations:** 1 College of Basic Medicine, Key Laboratory of Cellular Physiology, Ministry of Education, Shanxi Medical University 74648, Taiyuan, Shanxi, China; 2 Key Laboratory of Bio-Resources and Eco-Environment, Animal Disease Prevention and Green Development Key Laboratory of Sichuan Province, College of Life Science, Sichuan University 12530, Chengdu, China; 3 Department of Biochemistry and Molecular Biology, College of Basic Medicine, Shanxi Key Laboratory of Birth Defect and Cell Regeneration, MOE Key Laboratory of Coal Environmental Pathogenicity and Prevention, Shanxi Medical University 74648, Taiyuan, China; 4 Datong Center For Disease Control and Prevention, Datong, China; Shandong First Medical University, Jinan, Shandong, China

**Keywords:** RND3, IKKε, encephalomyocarditis virus, ubiquitination, interferon

## Abstract

Encephalomyocarditis virus (EMCV), one of the most important picornaviruses, infects many mammalian species and causes encephalitis, myocarditis, neurologic diseases, and diabetes, but the host factors that restrict infection and replication of the virus remain poorly understood. RND3, a member of the Rho GTPase family, is involved in the regulation of actin cytoskeleton dynamics, migration, and proliferation; however, its role in antiviral innate immunity is not clear. Herein, we revealed that the host restriction factor for EMCV replication, RND3, positively regulates type I interferon (IFN-I) induction. We showed that RND3 enhances the production of IFN-β and IFN-stimulated genes, leading to the attenuation of EMCV propagation. Mechanistically, RND3 specifically interacts with IκB kinase epsilon (IKK**ε**) and promotes the TRIM21-mediated K63-linked ubiquitination of IKK**ε**. However, RND3 expression is inhibited by EMCV, establishing a negative regulatory feedback loop. Taken together, these findings provide insight into the molecular mechanisms by which RND3 promotes IFN-I production by targeting IKKε to inhibit viral replication.

**IMPORTANCE:**

Host cell restriction factors perform key antiviral functions during viral infection. Herein, we demonstrate that RND3 is a host restriction factor in EMCV infection that plays a role in the antiviral signaling pathway. We found that RND3 is involved in the type I IFN pathway by interacting with IKKε, which negatively regulates EMCV transmission. However, EMCV infection effectively reduces RND3 expression in cells. Collectively, these results reveal the role of RND3 in the IFN pathway and identify potential targets for controlling EMCV infection.

## INTRODUCTION

Encephalomyocarditis virus (EMCV), a member of the Picornaviridae and an important zoonotic virus, is a small nonenveloped single-strand RNA virus (1). The EMCV genome is approximately 7.8 kb long and contains a unique open reading frame (ORF) and 5′ and 3′ untranslated regions. The ORF encodes four structural proteins (VP4, VP2, VP3, and VP1) and nine nonstructural proteins (Lpro, 2A, 2B, 2B*, 2C, 3A, 3B, 3C, and 3D) (2). The main manifestations of EMCV infection are encephalitis, myocarditis, and type 1 diabetes (1, 3). EMCV has a global distribution and a wide host range and has been isolated from a wide range of wildlife, including pigs, wild boars, elephants, dogs, rats, and primates, as well as humans, suggesting that the virus poses potential public health and safety risks (4–8).

The innate immune system responds rapidly to a variety of invading pathogens and plays an important role in early clearance by the host (9). During viral invasion into the host, the viral genome produces some conserved components, known as pathogen-associated molecular patterns (PAMPs), which can be recognized by pattern recognition receptors (PRRs). The major classes of cellular PRRs have been identified: Toll-like receptors (TLRs), RIG-I-like receptors (RLRs), NOD-like receptors (NLRs), DNA-dependent activator of interferon-regulatory factors (DAI), and Absent In Melanoma 2 (AIM2) (10). In RIG-I-mediated signaling, RIG-I and MDA5 are in a state of self-repression when the cell is in a resting state (10, 11). Upon viral infection, the recognition of viral PAMPs by host RLRs results in a conformational change mediated by the ATPase/deconjugate enzyme, leading to CARD exposure (12). RIG-I and MDA5 are subsequently translocated intracellularly to and bind to MAVS (also known as IPS-1/VISA), which is localized on mitochondria (11). The above interaction results in the activation of downstream protein kinases via the TNF receptor-associated factors tripartite motif protein 3 (TRAF3), TRAF6, IκB kinase (inhibitor of nuclear factor kappa-B kinase [IKK] family, including IκB kinase complex epsilon [IKKε]), and TANK-binding kinase 1 (TBK1) (13). Furthermore, the phosphorylation of IKKε and TBK1 activates downstream transcription factors, including interferon regulatory factors 3 and 7 (IRF3/IRF7), to translocate, enter the nucleus, and bind to the interferon-stimulated response element (ISRE), which then induces type I IFN production and the downstream production of antiviral proteins (14).

RND3 belongs to the atypical RND subfamily of Ras homologous (Rho) guanosine triphosphatases (GTPases) (the Rho GTPase family) (15). Most GTPases function by molecularly switching between two conformations: the active GTP-bound state and the inactive GDP-bound state (16). RND proteins are unique in that they have a GTPase structure and the ability to bind nucleotides but lack the GTPase activity to switch from a GTP-bound to a GDP-bound conformation (17). Previous studies have revealed that RND3 functions in response to RhoA/Rho-associated coiled-coil kinase 1 (ROCK1) signaling, which is involved in the regulation of a variety of cellular features and processes, such as the cytoskeleton, the cell cycle, vesicle trafficking, proliferation, and survival (16). Research has shown that the overexpression of RND3 inhibits chikungunya virus (CHIKV) replication (18). However, the function of RND3 in RLR-mediated innate immunity and EMCV replication has not been thoroughly investigated.

In this study, through screening analyses, we found that RND3 is a restrict factor for host anti-EMCV infection. We characterized the key functions of RND3 that participate in EMCV replication. We hypothesized that RND3 target IKKε protein to modulate IFN-β production, ultimately inhibiting virus proliferation. However, RND3 protein expression was negatively regulated after EMCV infection. Our data revealed that RND3 acts as a positive modulatory molecule of IFN-I, which suggests a novel role for RND3 in innate immunity and in combating EMCV infections.

## MATERIALS AND METHODS

### Cells, Viruses, and Plasmids

HEK293T was cultured in Dulbecco’s modified Eagle’s medium (DMEM, Cellmax, China) supplemented with 10% fetal bovine serum (FBS, Cellmax, China) and 100 IU/mL penicillin‒streptomycin solution (Cellmax, China). The primary C57BL/6 mouse embryo fibroblasts (MEFs) were prepared by oneself and cultured in DMEM supplemented with 10% FBS and 1% penicillin and streptomycin. All cells were cultured in an incubator at 37°C and 5% CO_2_. Recombinant vesicular stomatitis virus expressing green fluorescent protein (VSV-GFP) and Sendai virus (SeV) was kindly provided by Prof. Xin Cao (Jilin Agricultural University, China). Viruses were inoculated into and propagated in 9-day-old specific-pathogen-free (SPF) chicken embryos for 3 days and then harvested and stored at −80˚C until use.

Full-length Homo sapiens IFI44 (NM_006417.5), ZFP36 (NM_003407.5), ZDHHC1 (NM_013304.3), and NEURL3 (NM_001285485.2) were amplified from HEK293T cells and cloned into pcDNA3.1-Myc. Full-length human RND3 (NM_001254738.1) and Mus musculus RND3 (NM_028810.2) were amplified from HEK293T cells and MEF cells and then cloned into pcDNA3.1-Myc, pcDNA3.1-Flag, and pEGFP-C1 to generate pcDNA3.1-Myc-hRND3/mRND3, pcDNA3.1-Flag-hRND3/mRND3, and pEGFP-hRND3. HA-MDA5, HA-RIG-I, HA-MAVS, HA-TBK1, HA-IKKε, HA-IRF3, Flag-TRIM21, HA-TRIM21 was purchased from Miaoling Biotechnology Co. (China).

### Antibodies

RND3 Monoclonal antibody (66228-1-Ig), Beta Actin Monoclonal antibody (66009-1-Ig), MYC tag Polyclonal antibody (16286-1-AP), HA tag Polyclonal antibody (51064-2-AP), DYKDDDDK tag Polyclonal antibody (20543-1-AP), His-tag Polyclonal antibody (10001-0-AP), CoraLite Plus 647-Goat Anti-Rabbit Recombinant Secondary Antibody purchased from Proteintech Co. (China). Mouse Anti-Rabbit IgG LCS (A25022), Goat Anti-Rabbit IgG HCS (A25222) purchased from Abbkine Scientific Co. (China). IRF3 antibody (AF2485), p-IRF3 antibody (AF1594), RIG-I antibody (AG4872), MAVS antibody (AF7425), TBK1 antibody (AF8103), Cy3-labeled goat anti-mouse IgG (A0521), Dylight 405-conjugated goat anti-rabbit IgG (A0605), and Dylight 405-conjugated goat anti-mouse IgG (A0609) secondary antibodies purchased from Beyotime Biotech (China). IKKε antibody (2690T) purchased from Cell Signaling Technology (USA).

### Transfection and dual-luciferase reporter assays

The HEK293T cells were plated in 24-well plates (1 × 10^5^ cell/mL) for 24 h. Then, co-transfected with Luciferase reporter plasmids IFN-β-Luc or ISRE-Luc, and pRL-TK plasmid, along with the indicated amount of control plasmid or plasmids expressing RND3 used Lipofectamine 8000 (Beyotime Biotech, Beijing, China) with the indicated amount of expression construct according to the manufacturer’s instructions. At 24 h post-transfection, the cells were left untreated or were treated with, poly(I:C), or SeV for another 12 h. Then, using dual-luciferase assays, the whole-cell extracts were prepared for examination. Activities of the reporter genes, such as firefly luciferase and renilla luciferase, were measured using a dual-luciferase reporter 100 assay system (Beyotime, Beijing, China), as directed by the manufacturer.

### VSV-GFP bioassay

HEK293T cells were grown in 24-well plates and transfected with the RND3 plasmids 300 ng, 500 ng. At 24 h post-transfection, the cells were then infected at an MOI of 0.01 with VSV-GFP. At 12 h post-infection, VSV-GFP replication was visualized by monitoring the GFP expression level by fluorescence microscopy.

### RNA interference assay

To investigate the function of RND3, we silenced its expression through siRNA. All siRNAs used in this study were designed and synthesized by Sangon Biotech (Sangon Biotech, China) to target RND3 coding region. The target siRNA sequence was as follows: siRNA-ctrl F:UUCUCC GAACGUGUCACGUTT, siRNA-ctrl R:ACGUGACACGUUCGGAGAATT. siRNA-1# F:CAGAACGUGAAAUGCAAGAUATT, siRNA-1# R:UAUCUUGCAUUUCACGUUCUGTT. siRNA-2# F:GCGGACAGAUGUUAGUACAUUTT, siRNA-2# R:AAUGUACUAACAUCUGUCCGCTT. siRNA-3# F:GCUCAGCUUUACAGUCGGAAATT, siRNA-3# R:UUUCCGACUGUAAAGCUGAGCTT. To analyze the effect of RND3 siRNA, HEK293T cells were seeded in 6-well plates at 50% confluency. Cells were transfected with siRND3-ctrl, siRND3-1, siRND3-2, or siRND3-3 (100 pmol/well) using a Lipofectamine 8000. The treated cells were incubated for 48 h. Then, cells were harvested and detected by western blotting or quantitative real-time polymerase chain reaction (qRT-PCR) assay.

### Confocal fluorescence microscopy

The cells were seeded on glass coverslips in 35-mm cell culture dishes and cultured overnight. Thereafter, the Hela cells were co-transfected with pEGFP-C1-RND3 and pcDNA3.1-mcherry-IKKε. After 24 h, the cells were washed three times with cold phosphate buffered saline (PBS) and fixed with 4% paraformaldehyde for 15 min at room temperature. Subsequently, the cells were incubated with DAPI at 37°C for 10 min and washed with cold PBS. Finally, images were captured using a laser scanning confocal microscope (Olympus FV3000).

### Co-immunoprecipitation assays

The HEK293T cells (1 *×* 10^5^ cells/mL) cultured in 24-well plates were co-transfected with the following plasmids: HA-MDA5, HA-RIG-I, HA-MAVS, HA-TBK1, HA-IKKε, HA-IRF3, or pcDNA3.1-Flag-RND3 using Lipofectamine 8000. The transfected cells were lysed on ice for 30 min after being transfected for 24 h with RIPA lysis solution (500 µL/well) containing 1 mM PMSF (Beyotime). The lysates were centrifuged for 30 min at 12,000 *g*. Approximately 25% of the supernatant was subjected to input assays, and the remaining supernatant was utilized for the co-immunoprecipitation (Co-IP) test with an anti-Flag agarose affinity gel (Beyotime). The agarose affinity gel (50 µL) was then centrifuged for 30 s at 4 ℃ to remove the solution before being rinsed with cold TBS. On a revolving platform, the agarose affinity gel was added to the cell lysate while being gently rocked at 4 ℃ overnight. The protein samples were analyzed using Western blotting after the agarose affinity gel was washed with cold TBS.

### Protein extraction and western blot

Cultured cells were harvested and lysed by RIPA lysis buffer. The SDS-PAGE sample loading buffer was added, and the samples were heated for 5 min at 95°C. The samples were subjected to 10% SDS-PAGE at 120 V for 100 min. The protein was transferred to a PVDF membrane (pore size, 0.22 mM; Beyotime) for 1.5 h at 200 mA. Afterward, the membrane was blocked with a blocking buffer containing 1% BSA for 1 h at room temperature. It was then incubated overnight at 4 ℃ with a primary antibody (1:1,000 dilution). The membrane was then washed three times and subsequently incubated with a secondary antibody (1:2,000 dilution) for 1 h at room temperature. After three washes, protein bands were detected using BeyoECL Moon chemiluminescent system (Beyotime).

### RNA extraction and quantitative real-time polymerase chain reaction

Total RNA from the samples was isolated using the RNA-easy isolation reagent (Vazyme Biotech Co., Ltd., R701, China) according to the manufacturer’s instruction. The RNA (1 μg) was further processed using RevertAid First Strand cDNA Synthesis kit (Thermo Fisher, USA) to produce cDNA in accordance with the manufacturer’s instructions. qRT-PCR was performed using a Bio-Rad system (Hercules, CA). ChamQ SYBR qPCR Master Mix (Vazyme Biotech, Nanjing, China) and gene-specific primers were used in a 20-μL volume. The samples were heated to 95°C for 10 min, followed by 40 cycles of PCR involving 10 s at 95°C, 20 s at 60°C, and 15 s at 72°C. The glyceraldehyde-3-phosphate dehydrogenase gene (GAPDH) was used as a housekeeping gene to normalize (relative) gene expression using the 2^−∆∆CT^ formula. The qRT-PCR primers used in this study are listed in Table 1.

**TABLE 1:** Primers used in this study

Primers	Forward (5′–3′)	Reverse (5′–3′)
IFN-β	GCTTGGATTCCTACAAAGAAGCA	ATAGATGGTCAATGCGGCGTC
CXCL10	GTGGCATTCAAGGAGTACCTC	TGATGGCCTTCGATTCTGGATT
RANTES	GGCAGCCCTCGCTGTCATCC	GCAGCAGGGTGTGGTGTCCG
OASL	CTGATGCAGGAACTGTATAGCAC	CACAGCGTCTAGCACCTCTT
ISG56	TCATCAGGTCAAGGATAGTC	CCACACTGTATTTGGTGTCTAGG
EMCV	CCCGACCTCTGCTAAGATACTAAC	GGGACTGGACCTATCATAGAAG
VSV	TGATACAGTACAATTATTTTGGGAC	GAGACTTTCTGTTACGGGATCTGG
GAPDH	CTCTGGTAAAGTGGATATTGT	GGTGGAATCATATTGGAACA

### Statistical analysis

Statistical analysis was performed using GraphPad Prism 5 software (Mann‒Whitney test, one-way; GraphPad Prism software, GraphPad Software Inc., La Jolla, CA). Statistical significance was set at *P* < 0.05.

## RESULTS

### Screening of restrictive factors against EMCV *in vitro*

**Fig 1:**
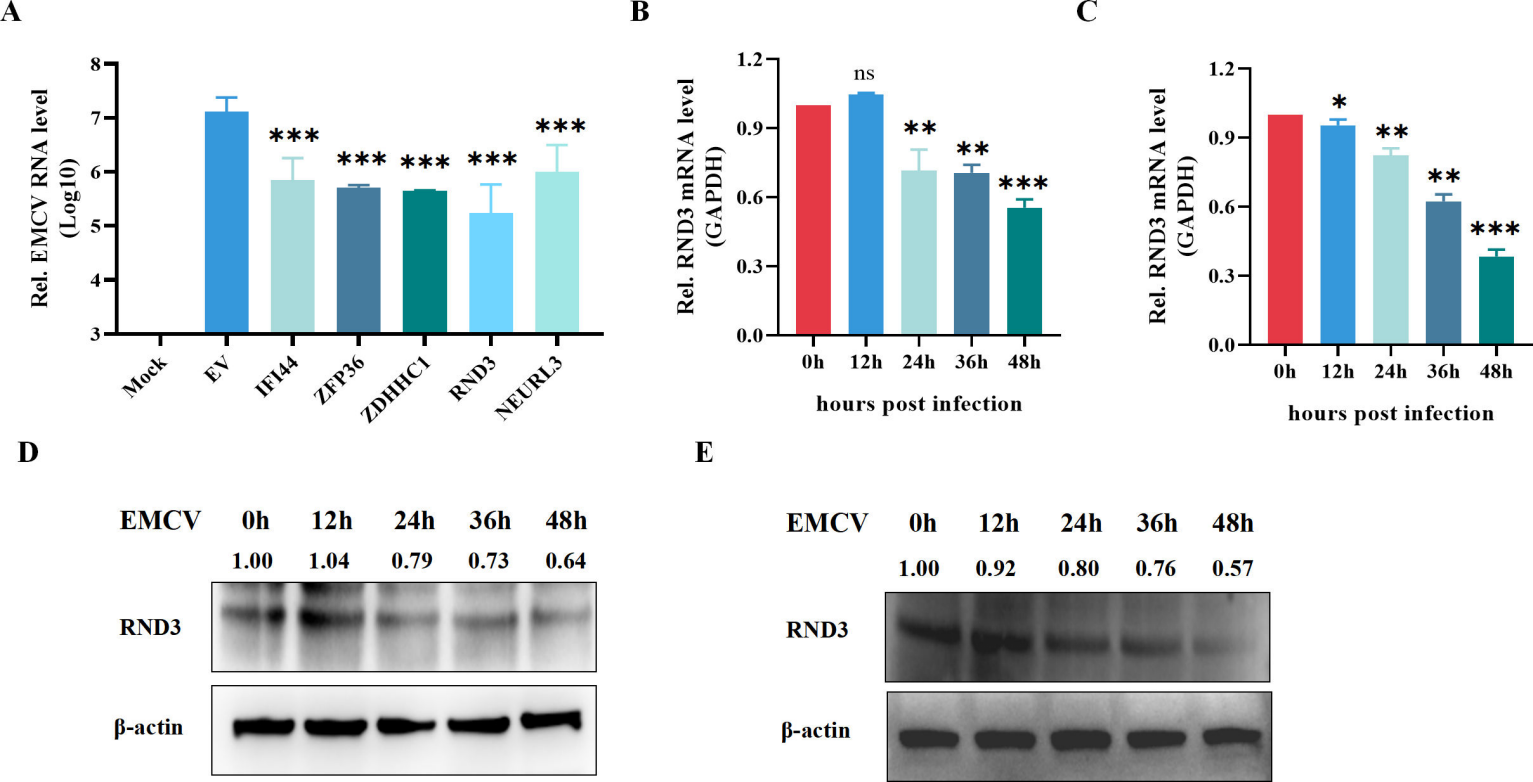
Screening of restrictive factors against EMCV *in vitro*. (**A**) HEK293T cells were transfected with plasmids pcDNA3.1-Myc-IFI44 (1 μg), pcDNA3.1-Myc-ZFP36 (1 μg), pcDNA3.1-Myc-ZDHHC1 (1 μg), pcDNA3.1-Myc-NEURL3 (1 μg), and pcDNA3.1-Myc-RND3 (1 μg) for 24 h. Then infected with EMCV at MOI = 0.1 and harvested cells after 24 h, qRT-PCR detected EMCV-VP1 gene mRNA. (**B–E**) HEK293T cells infected with different strains of EMCV at MOI = 0.1 were harvested after 0 h, 12 h, 24 h, 36 h, and 48 h, qRT-PCR detected RND3 mRNA. Protein level measured using anti-RND3 antibodies. β-actin was used as a loading control. The results are presented as means ± standard deviations. ns, not significant; *, *P* < 0.05; **, *P* < 0.01; ***, *P* < 0.001 vs control.

To screen antiviral genes with antiviral activity, we generated five gene expression plasmids with Myc-tags. IFI44, ZFP36, ZDHHC1, RND3, and NEURL3 were overexpressed in HEK293T cells and significantly reduced EMCV mRNA levels. Interestingly, Rho family GTPase 3 (RND3), a small GTP‐binding protein in the Rho family, had the highest inhibitory effect against EMCV replication (Fig. 1A). We found that different strains of EMCV infection significantly reduced RND3 expression (Fig. 1B and C). Additionally, western blotting revealed that different strains of EMCV infection weakened RND3 protein expression similar to the changes observed at the transcription level (Fig. 1C and D). These results indicate that RND3 is a potential limiting factor for the inhibition of viral replication.

### RND3 inhibits EMCV replication

**Fig 2:**
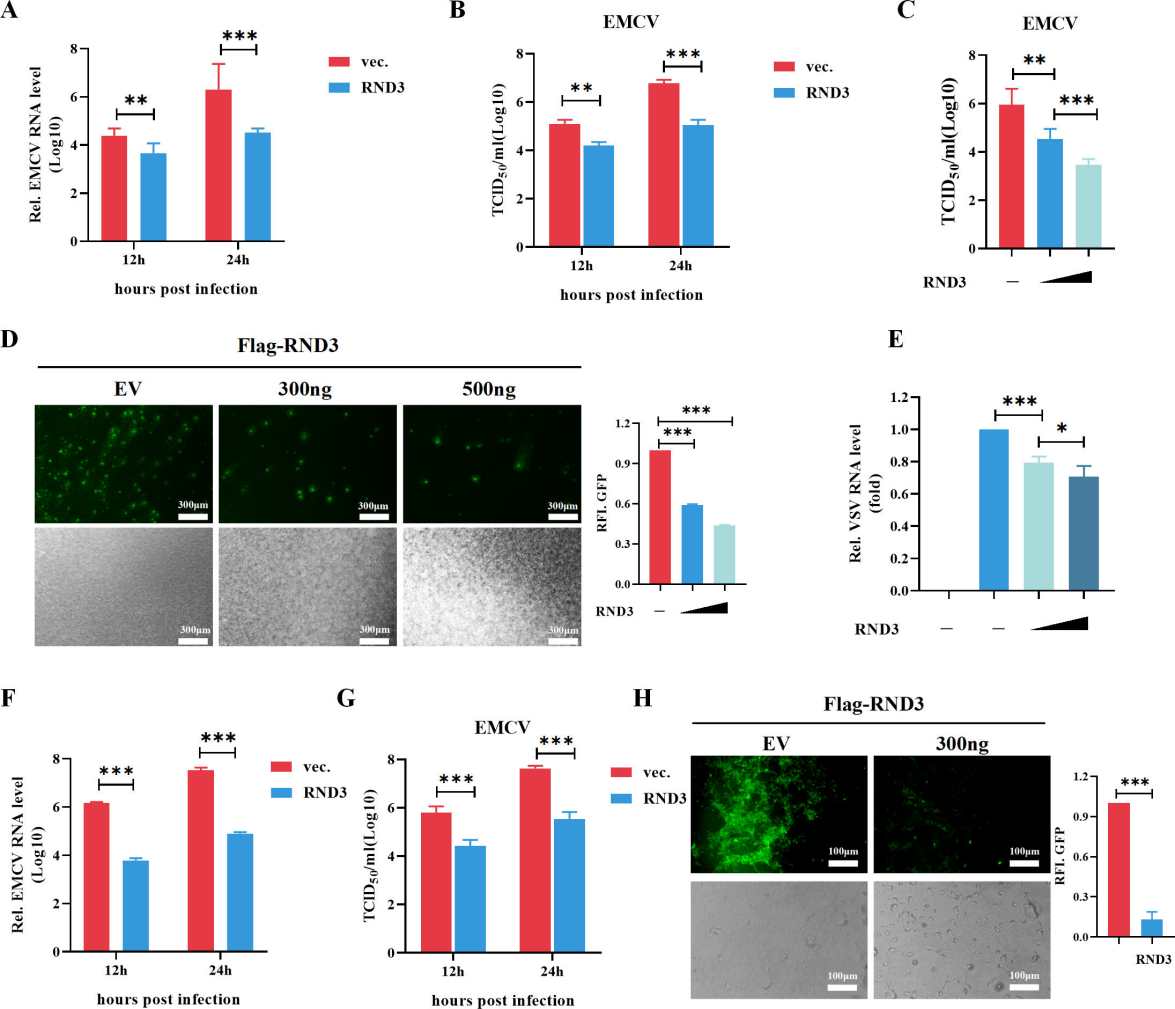
Overexpression of RND3 inhibits virus replication. (**A**) HEK293T cells were transfected with pcDNA3.1-Myc-hRND3 (1 μg) or pcDNA3.1-Myc (1 μg) for 24 h and then infected with EMCV at MOI = 0.1 were harvested at different points in time, qRT-PCR detected EMCV-VP1 gene mRNA. (**B**) Virus titers in the supernatants were analyzed by a TCID_50_ assay. (**C**) HEK293T cells were transfected with pcDNA3.1-Myc-hRND3 (1 μg), pcDNA3.1-Myc-hRND3 (2 μg), or pcDNA3.1-Myc (1 μg) for 24 h and then infected with EMCV at MOI = 0.1 were harvested at 24 post hours infection, virus titers in the supernatants were analyzed by a TCID_50_ assay. (**D**) HEK293T cells were transfected with pcDNA3.1-Myc-hRND3 (300 ng), pcDNA3.1-Myc-hRND3 (500 ng), or pcDNA3.1-Myc (300 ng) for 24 h, the cells then were infected with VSV-GFP at MOI = 0.01 for 18 h, the cells were observed microscopically. (**E**) VSV mRNA level was detected by qRT-PCR. (**F**) MEF cells were transfected with pcDNA3.1-Myc-mRND3 (1 μg) or pcDNA3.1-Myc (1 μg) for 24 h and then infected with EMCV at MOI = 0.1 were harvested at different points in time, qRT-PCR detect EMCV-VP1 gene mRNA. (**G**) Virus titers in the supernatants were analyzed by a TCID_50_ assay. (**H**) MEF cells were transfected with pcDNA3.1-Myc-mRND3 (300 ng) or pcDNA3.1-Myc (300 ng) for 24 h, the cells then were infected with VSV-GFP at MOI = 0.01 for 18 h, the cells were observed microscopically. The results are presented as means ± standard deviations. *, *P* < 0.05; **, *P* < 0.01; ***, *P* < 0.001 vs control.

RND3 expression decreased during EMCV infection, suggesting that RND3 might perform a pivotal function in viral infections. To further investigate the role of RND3 in virus replication, we overexpressed RND3 in cells, followed by EMCV infection. Compared with the empty vector, RND3 overexpression significantly inhibited viral replication at different times during EMCV infection (Fig. 2A). The TCID_50_ significantly decreased after RND3 overexpression (Fig. 2B). Additionally, RND3 inhibited EMCV replication in a dose-dependent manner (Fig. 2C). Furthermore, recombinant vesicular stomatitis virus expressing green fluorescent protein (VSV-GFP) was introduced into cells to assess VSV replication via fluorescence microscopy. The relative fluorescence intensity (RFI) of GFP^+^ was significantly repressed in HEK293T cells transfected with increasing amounts of exogenous RND3 plasmids (Fig. 2D and E). Furthermore, the antiviral effect of RND3 was tested using primary mouse embryo fibroblasts (MEFs). As the results showed that overexpression of RND3 significantly inhibited EMCV replication and decreased the virus titers (Fig. 2F and G). Additionally, RND3 effectively suppressed the fluorescence level of VSV-GFP in MEFs (Fig. 2H). Taken together, these findings indicate that RND3 is a critical antiviral protein in a cellular context.

### RND3 activates virus-triggered IFN-β production

**Fig 3:**
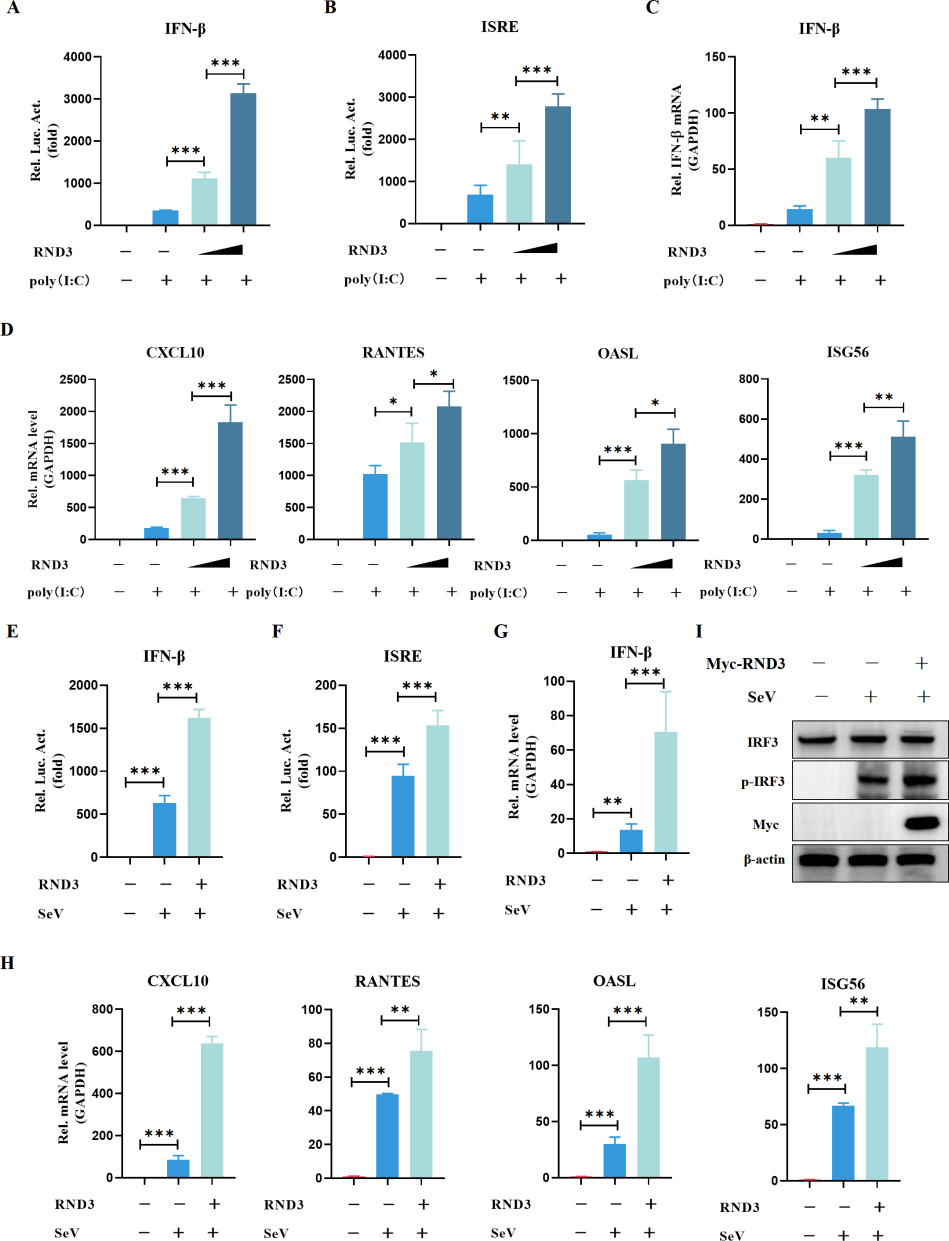
RND3 activates type I interferon production. (**A and B**) HEK293T cells were co‐transfected with pcDNA3.1‐Myc‐RND3 (0.5 μg), pcDNA3.1‐Myc‐RND3 (1 μg), pIFN-β‐luc (200 ng), pISRE‐luc (200 ng), and pRL‐TK (10 ng). After 24 h, the cells were stimulated with poly(I:C) for 12 h. Then, luminescence was detected using dual‐luciferase report. (**C and D**) HEK293T cells were co‐transfected with pcDNA3.1‐Myc‐RND3 (0.5 μg), pcDNA3.1‐Myc‐RND3 (1 μg), or pcDNA3.1-Myc (1 μg). After 24 h, the cells were stimulated with poly(I:C) for 12 h. Then, *IFN-β, CXCL10, RANTES, OASL,* and *ISG56* mRNA levels were detected using qRT-PCR. (**E and F**) HEK293T cells were co‐transfected with pcDNA3.1‐Myc‐RND3 (0.5 μg), pIFN-β‐luc (200 ng), pISRE‐luc (200 ng), and pRL‐TK (10 ng). After 24 h, the cells were stimulated with SeV at MOI = 1 for 12 h. Then, luminescence was detected using dual‐luciferase report. (**G and H**) HEK293T cells were co‐transfected with pcDNA3.1‐Myc‐RND3 (0.5 μg), or pcDNA3.1-Myc (1 μg). After 24 h, the cells were stimulated with SeV at MOI = 1 for 12 h. Then, *IFN-β, CXCL10, RANTES, OASL,* and *ISG56* mRNA levels were detected using qRT-PCR. (**I**) HEK293T cells were transfected with pcDNA3.1‐Myc‐RND3 (1 μg) or pcDNA3.1-Myc (1 μg). Twenty hours after transfection, the cells were infected with SeV at MOI = 1 for the indicated times. The protein expression was analyzed by Western blotting with the indicated antibodies. β-Actin was used as a loading control. The results are presented as means ± standard deviations. *, *P* < 0.05; **, *P* < 0.01; ***, *P* < 0.001 vs control.

To explore whether RND3 plays a biological role in the production of interferon, HEK293T cells were transfected with IFN-β-Luc and ISRE-Luc in addition to RND3 expression plasmids and transfected with poly(I:C) (polyinosinic:polycytidylic acid), an RNA virus analog recognized by RIG-I. First, IFN-β promoter-regulated luciferase reporter activity was examined. The results revealed that RND3 overexpression significantly increased IFN-β (Fig. 3A) and ISRE (Fig. 3B) promoter activity in a dose-dependent manner in response to stimulation with poly(I:C). Also, the same result was observed in MEF cells, where overexpression of RND3 increased IFN-β (Fig. S1A) and ISRE (Fig. S1B) promoter activity. To investigate the influence of the RND3 protein on the host antiviral response, we next assessed the gene transcription levels of IFN-β and downstream IFN-stimulated genes (ISGs) in poly(I:C)-stimulated cells. RND3 overexpression significantly increased the gene transcription levels of *IFN-β, CXCL10, RANTES, OASL*, and *ISG56* stimulated with poly(I:C) (Fig. 3C and D). These results indicate that RND3 effectively enhanced poly(I:C)-induced IFN-β production. Hence, we hypothesized that RND3 affects RNA virus-induced IFN-I production. Then, we used an RNA virus (Sendai virus, SeV) to infect cells. RND3 overexpression significantly increased IFN-β (Fig. 3E and Fig. S1C) and ISRE (Fig. 3F and Fig. S1D) promoter activity, and the gene transcription levels of IFN-β (Fig. 3G), *CXCL10, RANTES, OASL*, and *ISG56* both in HEK293T cells and MEF cells infected with SeV (Fig. 3H and Fig. S1E). In addition, RND3 increased the SeV infection-induced phosphorylation of IRF3 (Fig. 3I), indicating further activation of the antiviral response. These results suggest that RND3 is an interferon regulatory gene in cells that activates the production of IFN-β and the expression of ISGs.

### Silencing RND3 attenuates intracellular antiviral immunity

**Fig 4:**
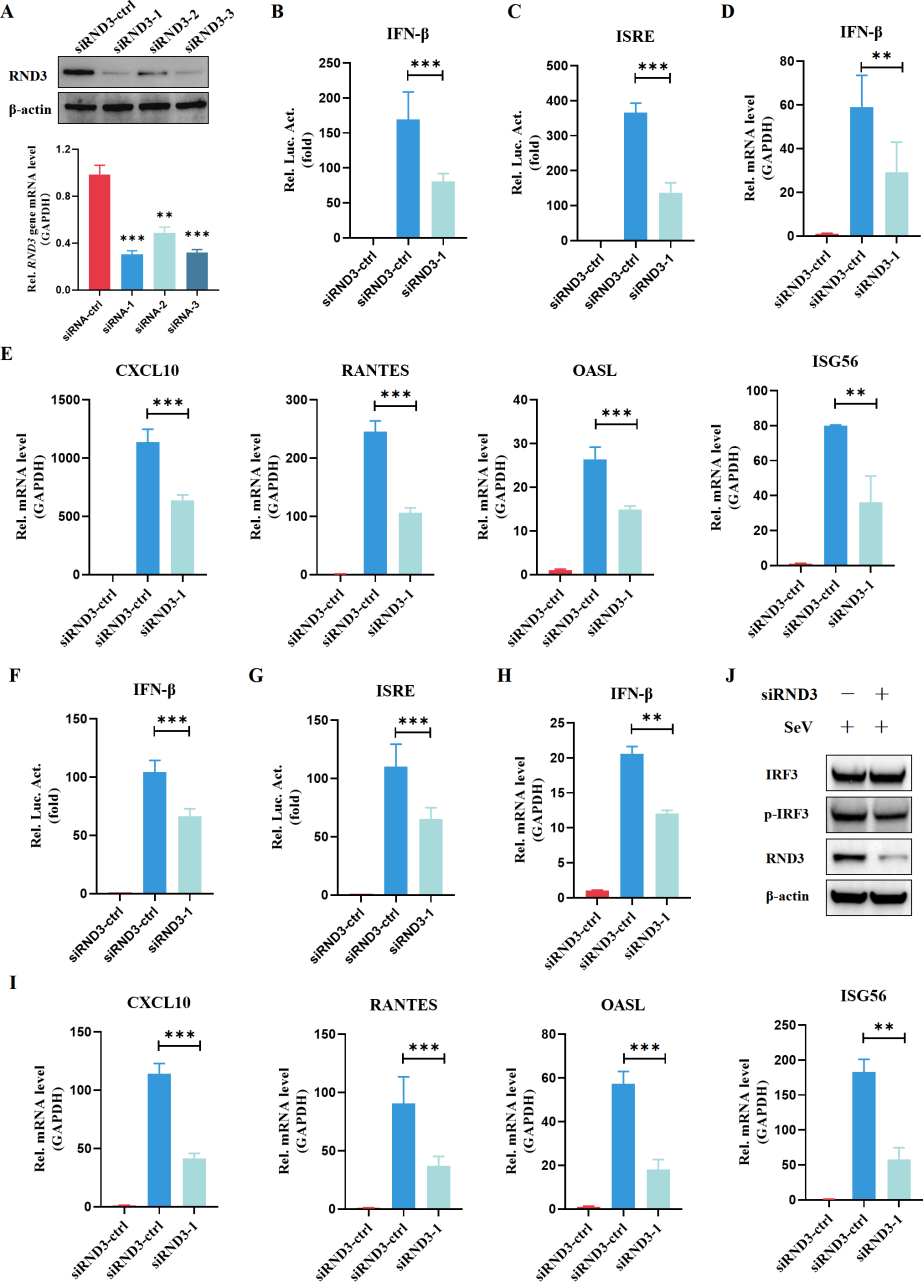
Silencing RND3 inhibits type I interferon production. (**A**) HEK293T cells were transfected with negative control siRNA (siRND3-ctrl) or siRND3 against RND3. The protein expression was analyzed by Western blotting with anti-RND3 and anti-β-actin antibodies. The mRNA of RND3 was quantified by qRT-PCR. (**B and C**) HEK293T cells were transfected with negative control siRNA (siRND3-ctrl), siRND3-1 for 24 h and then co‐transfected with pIFN-β‐luc (200 ng), pISRE‐luc (200 ng), and pRL‐TK (10 ng). After 24 h, the cells were stimulated with poly(I:C) for 12 h. Then, luminescence was detected using dual‐luciferase report. (**D and E**) HEK293T cells were transfected with negative control siRNA (siRND3-ctrl), siRND3-1 for 48 h. The cells were stimulated with poly(I:C) for 12 h. Then, *IFN-β, CXCL10, RANTES, OASL,* and *ISG56* mRNA levels were detected using qRT-PCR. (**F and G**) HEK293T cells were transfected with negative control siRNA (siRND3-ctrl), siRND3-1 for 24 h and then co‐transfected with pIFN-β‐luc (200 ng), pISRE‐luc (200 ng), and pRL‐TK (10 ng). After 24 h, the cells were stimulated with SeV at MOI = 1 for 12 h. Then, luminescence was detected using a dual‐luciferase report. (**H and I**) HEK293T cells were transfected with negative control siRNA (siRND3-ctrl), siRND3-1 for 48 h. The cells were stimulated with with SeV at MOI = 1 for 12 h. Then, *IFN-β, CXCL10, RANTES, OASL,* and *ISG56* mRNA levels were detected using qRT-PCR. (**J**) HEK293T cells were transfected with negative control siRNA (siRND3-ctrl), siRND3-1 for 48 h. The cells were stimulated with with SeV at MOI = 1 for 12 h. The protein expression was analyzed by Western blotting with the indicated antibodies. β-Actin was used as a loading control. The results are presented as means ± standard deviations. **, *P* < 0.01; ***, *P* < 0.001 vs control.

To better understand the function of RND3, we used siRNA to knock down RND3 expression in HEK239T cells. siRND3-1 efficiently downregulated RND3 expression at both the mRNA and protein levels (Fig. 4A). To investigate the effects of RND3 on the RLR pathway, we treated cells with poly(I:C) and assessed the impact of RND3 knockdown on IFN-β production. Both IFN-β and ISRE promoter activities were significantly attenuated upon poly(I:C) (Fig. 4B and C) stimulation when RND3 was knocked down. Similarly, RND3 knockdown consistently resulted in a decrease in IFN-β production (Fig. 4D). In addition, the mRNA levels of downstream ISGs, including *CXCL10, RANTES, OASL,* and *ISG56*, were reduced in poly(I:C)-stimulated cells (Fig. 4E). These results indicated that RND3 deficiency reduced poly(I:C)-induced IFN-β production. The role of SeV infection in IFN-β production in the absence of RND3 was explored further. SeV-induced IFN-β and ISRE promoter activity was significantly inhibited in RND3-knockdown cells (Fig. 4F and G). Additionally, the transcription of IFN-β (Fig. 4H) and ISGs (Fig. 4I) was attenuated. Importantly, the phosphorylation of the IRF3 protein in the RLR pathway decreased in the absence of RND3 (Fig. 4J). Collectively, these results suggest that RND3 deletion attenuates the RNA virus-induced IFN-β response.

### RND3 knockdown promotes viral replication in cells

**Fig 5:**
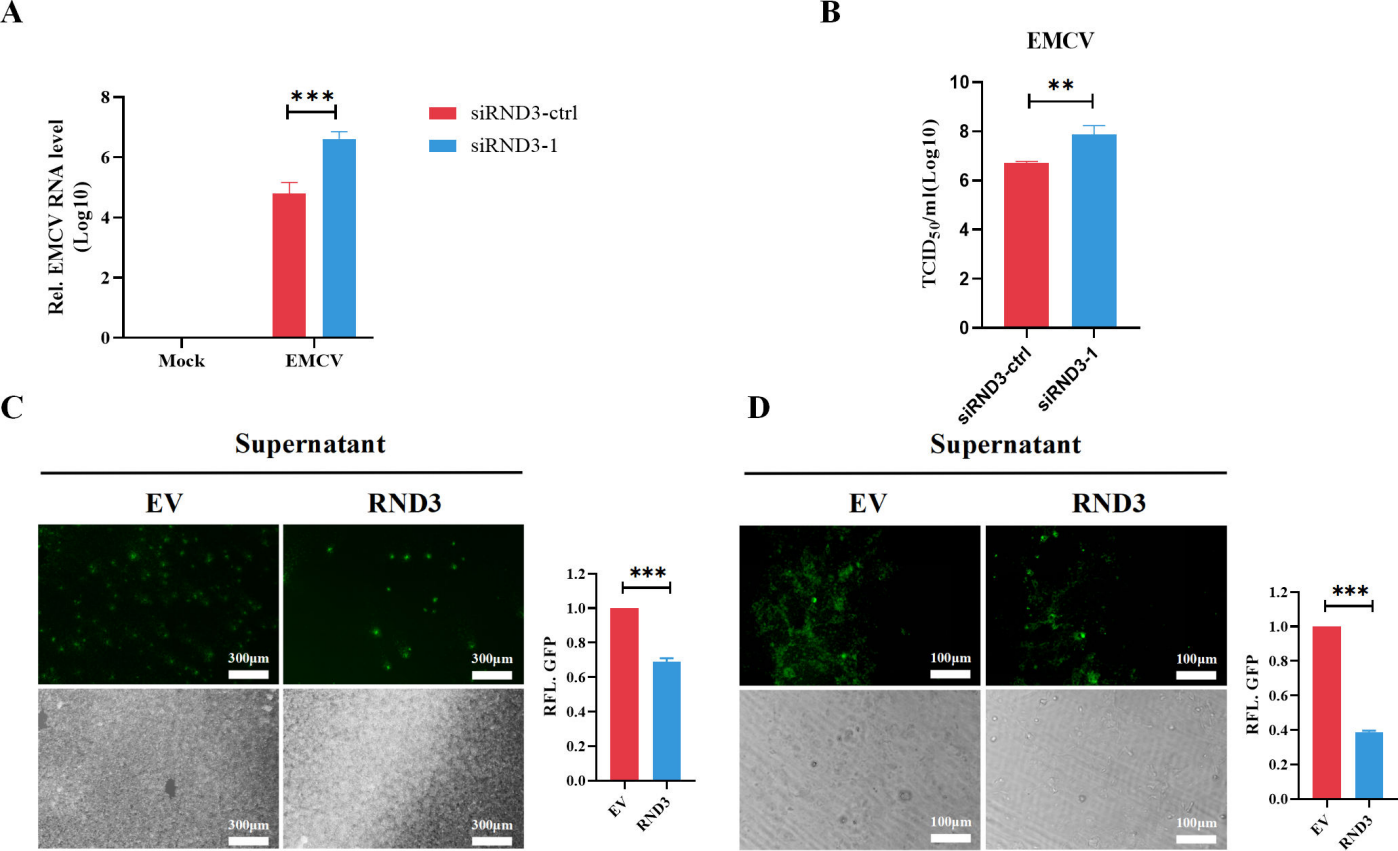
knockdown of RND3 promotes virus replication. (**A**) HEK293T cells were transfected with negative control siRNA (siRNA-ctrl), siRND3-1 for 48 h and then infected with EMCV at MOI = 0.1 were harvested at 24 post hours infection, qRT-PCR detected EMCV-VP1 gene mRNA. (**B**) Virus titers in the supernatants were analyzed by a TCID_50_ assay. (**C and D**) HEK293T or MEF cells were transfected with pcDNA3.1-Myc-hRND3/mRND3 (1 μg) or pcDNA3.1-Myc (1 μg) for 24 h, the cells then were infected with SeV (MOI = 1) for 24 h. The supernatants were inactivated by ultraviolet radiation and were collected to treat fresh HEK293T or MEF cells for another 24 h. The cells were infected with VSV-GFP (MOI = 0.01) for 18 h, the cells were observed microscopically. The results are presented as means ± standard deviations. **, *P* < 0.01; ***, *P* < 0.001 vs control.

To investigate the viral infection in the absence of RND3, we knocked down RND3 expression using siRNA. Subsequently, HEK293T cells were challenged with EMCV. Compared with siRNA-ctrl, siRND3-1 facilitated EMCV replication in cells (Fig. 5A). The TCID_50_ was significantly restored after siRNA-1 transfection (Fig. 5B). Next, we investigated whether RND3 plays a role in the expression of cellular antiviral effector genes. UV radiation-inactivated supernatant from RND3-transfected cells infected with SeV promoted the replication of VSV-GFP in HEK293T cells (Fig. 5C) and MEF cells (Fig. 5D). These results indicate that RND3 promotes the secretion of SeV-induced antiviral factors to inhibit virus replication.

### RND3 enhances the RLRs signaling pathway adaptor protein-mediated IFN-I response

**Fig 6:**
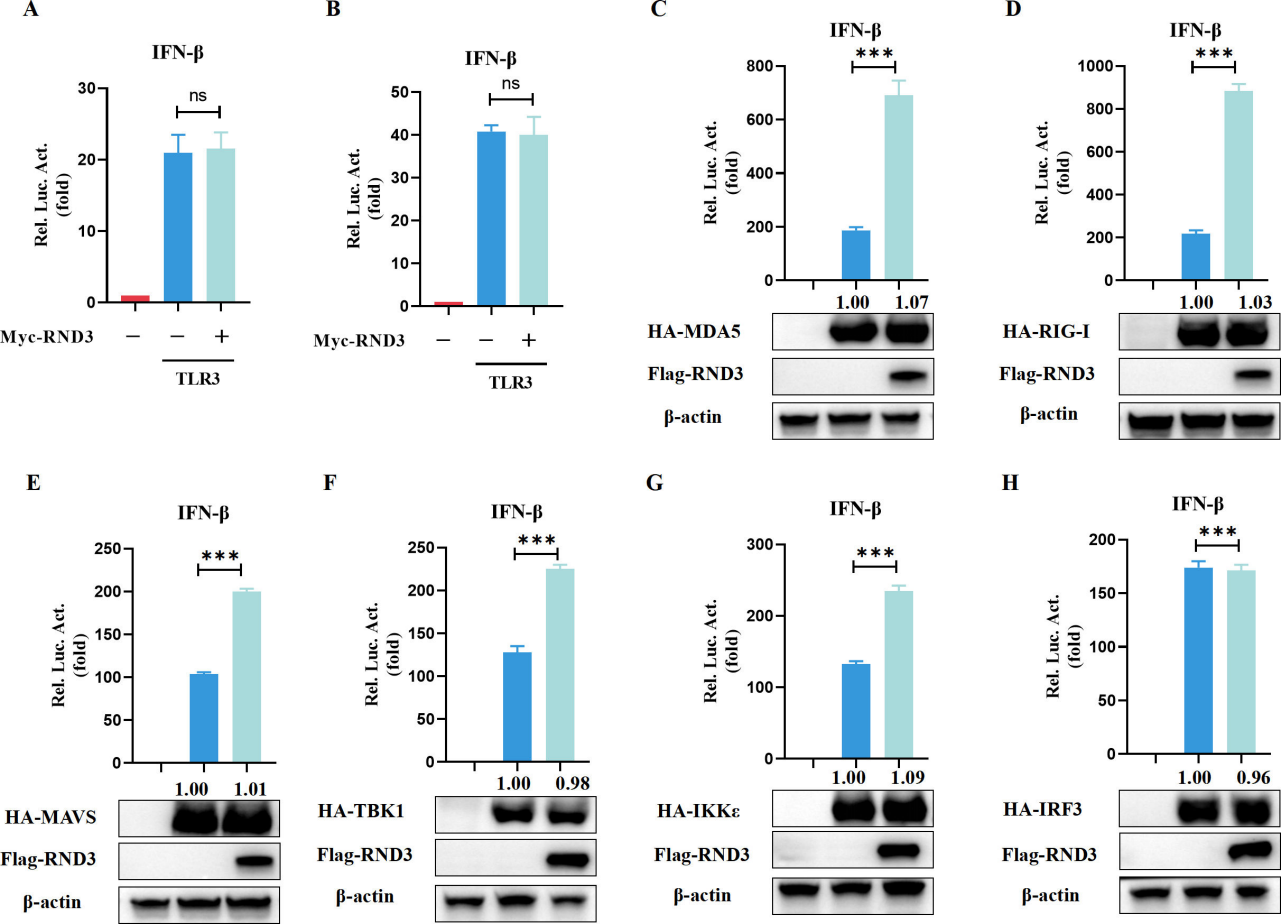
RND3 enhances adaptor protein mediated the IFN-I response. (**A and B**) HEK293T or MEFs cells were co-transfected with pcDNA3.1-Flag-hRND3/mRND3 (0.5 μg), pIFN-β‐luc (200 ng), pRL‐TK (10 ng), and TLR3 expressing plasmid for 24 h, followed by poly(I:C) treatment for 6 h, the luminescence was detected using dual‐luciferase report. (**C–H**) HEK293T cells were co-transfected with the following plasmids: pcDNA3.1-Flag-hRND3 (0.5 μg), pIFN-β‐luc (200 ng), pISRE‐luc (200 ng), and pRL‐TK (10 ng), HA-MDA5 (0.5 μg), HA-RIG-I (0.5 μg), HA-MAVS (0.5 μg), HA-TBK1 (0.5 μg), HA-IKKε (0.5 μg), or HA-IRF3 (0.5 μg). After 24 h, the luminescence was detected using dual‐luciferase report. The protein expression was analyzed by Western blotting with the indicated antibodies. β-Actin was used as a loading control. The results are presented as means ± standard deviations. ns, not significant; ***, *P* < 0.001 vs control.

We next sought to determine the molecular mechanisms by which RND3 promotes SeV-triggered IFN-I production. Viral RNA can activate TLRs and RLRs signaling pathways to mediate IFN production. However, HEK293T cells lack TLR3 expression and, thus, are defective in the TLR-mediated interferon signaling (19). To investigate the effect of RND3 on the TLR3-mediated IFN signaling, the cells were transfected with plasmids expressing TLR3, pIFN-β-luc, and Myc-RND3, followed by poly(I:C) treatment. The results showed that RND3 is not involved in regulating TLR3- mediated signaling pathways (Fig. 6A and B). To identify targets in the RLRs signaling pathway through which RND3 induced IFN-β activation, we transfected RND3-overexpressing cells with plasmids encoding MDA5, RIG-I, MAVS, TBK1, IKKε, or IRF3 driven by the IFN-β promoter. RND3 overexpression promoted IFN-β promoter activation triggered by MDA5 (Fig. 6C), RIG-I (Fig. 6D), MAVS (Fig. 6E), TBK1 (Fig. 6F), and IKKε (Fig. 6G) expression but not IRF3 (Fig. 6H) expression. Taken together, these data demonstrate that RND3 positively regulates the IFN-I response via the RLR signaling pathway.

### RND3 enhances the IFN-I response by targeting IKKε

**Fig 7:**
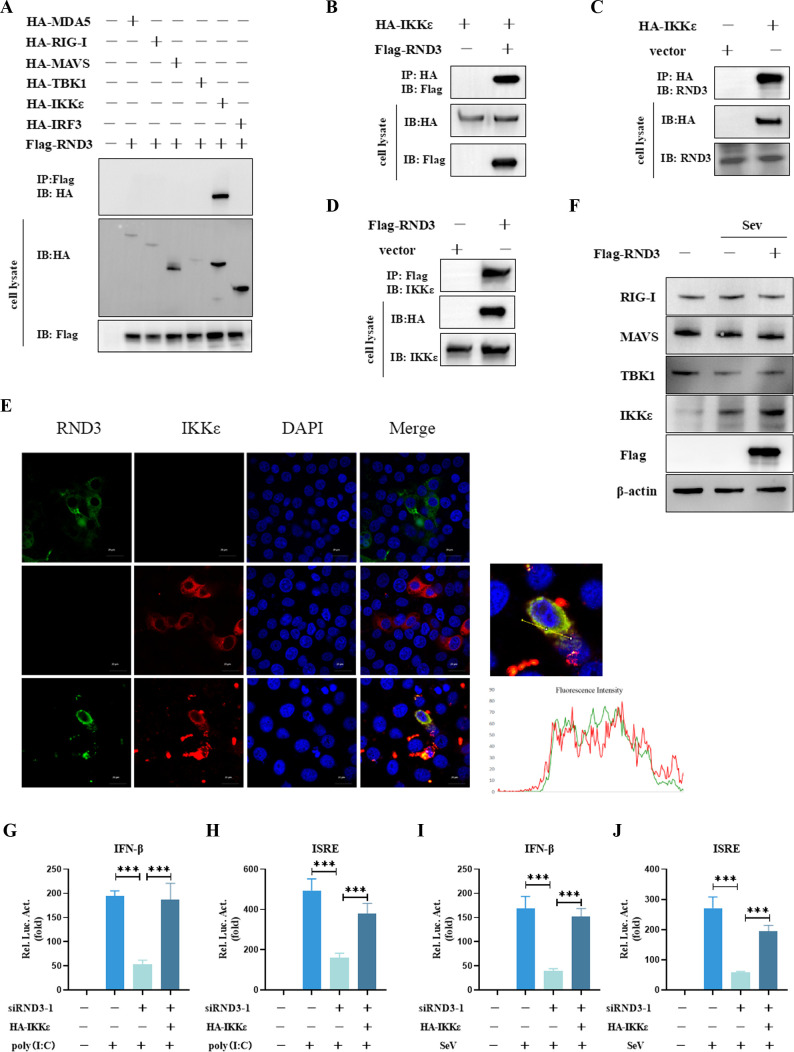
RND3-specific interaction with IKKε. (**A**) HEK293T cells were co-transfected with pcDNA3.1-Flag-RND3 (0.5 μg), HA-MDA5 (0.5 μg), HA-RIG-I (0.5 μg), HA-MAVS (0.5 μg), HA-TBK1 (0.5 μg), HA-IKKε (0.5 μg), or HA-IRF3 (0.5 μg). After 24 h, the cell lysate was harvested. Co-immunoprecipitation (Co-IP) was performed, and the precipitated proteins were analyzed by Western blotting using anti-HA antibodies. (**B**) HEK293T cells were co-transfected with pcDNA3.1-Flag-RND3 (0.5 μg) and HA-IKKε (0.5 μg). After 24 h, the cell lysate was harvested. Co-IP was performed, and the precipitated proteins were analyzed by Western blotting using anti-Flag antibodies. (**C and D**) HEK293T cells were transfected with HA-IKKε (0.5 μg) or pcDNA3.1-Flag-RND3 (0.5 μg). After 24 h, the cell lysate was harvested. Co-IP was performed and the precipitated proteins were analyzed by Western blotting using anti-RND3 or anti-IKKε antibodies. (**E**) HeLa cells were co-transfected with peGFP-C1-RND3 and pcDNA3.1-mcherry-IKKε. At 24 h post transfection, co-localization was performed using Confocal fluorescence microscopy. The nuclei were stained by DAPI. The fluorescence intensity profile of DAPI (blue), RND3 (green), and IKKε (red) was measured along the line drawn by Image J. (**F**) HEK293T cells were transfected with pcDNA3.1‐Myc‐RND3 (1 μg) or pcDNA3.1-Myc (1 μg). Twenty-four hours after transfection, the cells were infected with SeV at MOI = 1 for the indicated times. The protein expression was analyzed by Western blotting with the indicated antibodies. β-Actin was used as a loading control. (**G and H**) HEK293T cells were transfected with negative control siRNA (siRNA-ctrl), siRNA-1 for 24 h and then co‐transfected with HA-IKKε (0.5 μg), pIFN-β‐luc (200 ng), pISRE‐luc (200 ng), and pRL‐TK (10 ng). After 24 h, the cells were stimulated with poly(I:C) for 12 h. Then, luminescence was detected using dual‐luciferase report. (**I and J**) HEK293T cells were transfected with negative control siRNA (siRNA-ctrl), siRNA-1 for 24 h and then co‐transfected with HA-IKKε (0.5 μg), pIFN-β‐luc (200 ng), pISRE‐luc (200 ng), and pRL‐TK (10 ng). After 24 h, the cells were stimulated with SeV at MOI = 1 for 12 h. Then, luminescence was detected using a dual‐luciferase report. The results are presented as means ± standard deviations. ***, *P* < 0.001 vs control.

We hypothesized that RND3 interacts with the adaptor proteins of the RLRs signaling pathway. To test this hypothesis, we co-transfected RND3 with MDA5, RIG-I, MAVS, TBK1, IKKε, or IRF3 and performed coimmunoprecipitation (Co-IP) experiments. Co-IP experiments indicated that IKKε, but not the other tested adaptors, specifically interacted with RND3 (Fig. 7A). The interaction between the proteins was specific (Fig. 7B). Endogenous Co-IP experiments verified that IKKε bound with endogenous RND3 (Fig. 7C) and that RND3 bound with endogenous IKKε (Fig. 7D). Confocal microscopy confirmed that RND3 and IKKε colocalized in the cytoplasm (Fig. 7E). Consistent with the above results, RND3 overexpression significantly potentiated IKKε expression but not RIG-I, MAVS, TBK1, or IRF3 expression in virus-infected cells (Fig. 7F). To investigate the effect of IKKε in RND3-mediated IFN signaling, the cells were transfected with siRND3 and IKKε, followed by stimulating with poly(I:C) and SeV. The results showed that overexpression of IKKε effectively restored the activity of IFN-β and ISRE promoter after loss of RND3 (Fig. 7G through J). Collectively, these data demonstrate that IKKε is the specific target of the RND3 protein and that RND3 exerts antiviral activity by increasing IKKε.

### RND3 recruits TRIM21 to initiate the ubiquitination of IKKε

**Fig 8:**
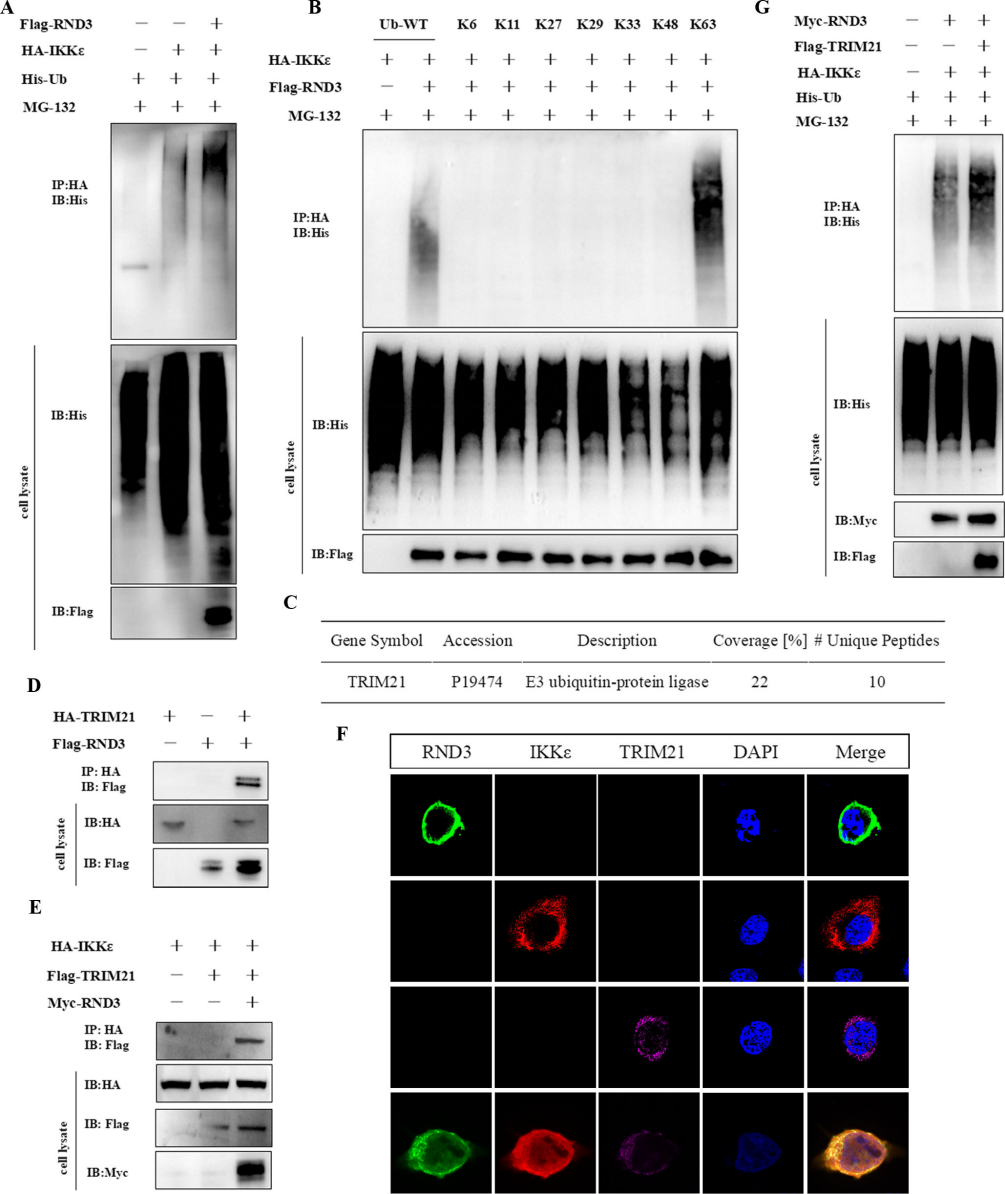
RND3 recruits TRIM21 to initiate the ubiquitination of IKKε. (**A**) HEK293T cells were co-transfected with pcDNA3.1-Flag-hRND3 (0.5 μg), HA-IKKε (0.5 μg), or His-Ub (0.2 μg). After 24 h, the cell lysate was harvested. Co-immunoprecipitation (Co-IP) was performed using an anti-HA antibody (MAb) (1:100). The precipitated proteins were analyzed by Western blotting using anti-His antibodies. (**B**) HEK293T cells were co-transfected with Flag-hRND3 (1 μg), HA-IKKε (1 μg), and His-Ub or the indicated Ub mutant plasmids (0.5 μg). After 24 h, the cell lysate was harvested. Co-IP was performed and the precipitated proteins were analyzed by Western blotting using anti-His antibodies. (**C**) HEK293T cells were co-transfected withpcDNA3.1-Flag-hRND3 (2 μg). After 24 h, the cell lysate was harvested. Immunoprecipitation was performed. Subsequently, the interaction proteins of RND3 in cells were analyzed by mass spectrometry. (**D**) HEK293T cells were transfected with HA-TRIM21 (1 μg) or pcDNA3.1-Flag-hRND3 (1 μg). After 24 h, the cell lysate was harvested. Co-immunoprecipitation (Co-IP) was performed using an anti-HA antibody (MAb) (1:100). The precipitated proteins were analyzed by Western blotting using anti-Flag antibodies. (**E**) HEK293T cells were transfected with pcDNA3.1-Myc-hRND3 (0.5 μg), HA-IKKε (0.5 μg), or Flag-TRIM21 (0.5 μg). After 12 h, the cell infected by EMCV at MOI = 0.01 was harvested after 12 h. Co-immunoprecipitation (Co-IP) was performed using an anti-HA antibody (MAb) (1:100). The precipitated proteins were analyzed by Western blotting using anti-Flag antibodies. (**F**) Hela cells were co-transfected with peGFP-C1-hRND3, Flag-TRIM21, and pcDNA3.1-mcherry-IKKε. At 24 h post-transfection, co-localization was performed using Confocal fluorescence microscopy. The nuclei were stained by DAPI. (**G**) HEK293T cells were co-transfected with pcDNA3.1-Myc-hRND3 (0.5 μg), HA-IKKε (0.5 μg), Flag-TRIM21 (0.5 μg), or His-Ub (0.2 μg). After 24 h, the cell lysate was harvested. Co-immunoprecipitation (Co-IP) was performed using an anti-HA antibody (MAb) (1:100). The precipitated proteins were analyzed by Western blotting using anti-His antibodies.

We co-transfected HEK293T cells with RND3, IKKε, and ubiquitin to perform an *in vitro* ubiquitin assay. The results demonstrated that RND3 catalyzed the ubiquitination of IKKε (Fig. 8A). To investigate the specific types of ubiquitination modifications of IKKε induced by RND3, we transfected with Flag-RND3, HA-IKKε, and various ubiquitin mutants plasmids, where only one lysine site (corresponding to the number indicated) was retained, while the remaining lysine sites were mutated. Overexpression of RND3 primarily induced K63-linked ubiquitination of IKKε (Fig. 8B). To investigate the mechanism of ubiquitination, we analyzed the proteins that interact with RND3 by IP-conjugated mass spectrometry; E3 ubiquitin ligase TRIM21 was identified as a potential protein that interacts with RND3 (Fig. 8C). Co-IP analysis demonstrated that TRIM21 interacted with RND3 (Fig. 8D). Furthermore, to verify the interaction between RND3, IKKε, and TRIM2, we co-transfected RND3, IKKε, and TRIM21 in cells. It was found that IKKε interacts with TRIM21 and forms a complex with RND3 (Fig. 8E) and colocalizes in the cytoplasm (Fig. 8F). Also, TRIM21 catalyzed the ubiquitination of IKKε (Fig. 8G). These results indicate that RND3 catalyzes the K63-linked ubiquitination of IKKε via TRIM21, leading to the activation of the IKKε-mediated IFN signaling pathway and subsequent antiviral effects.

## DISCUSSION

**Fig 9:**
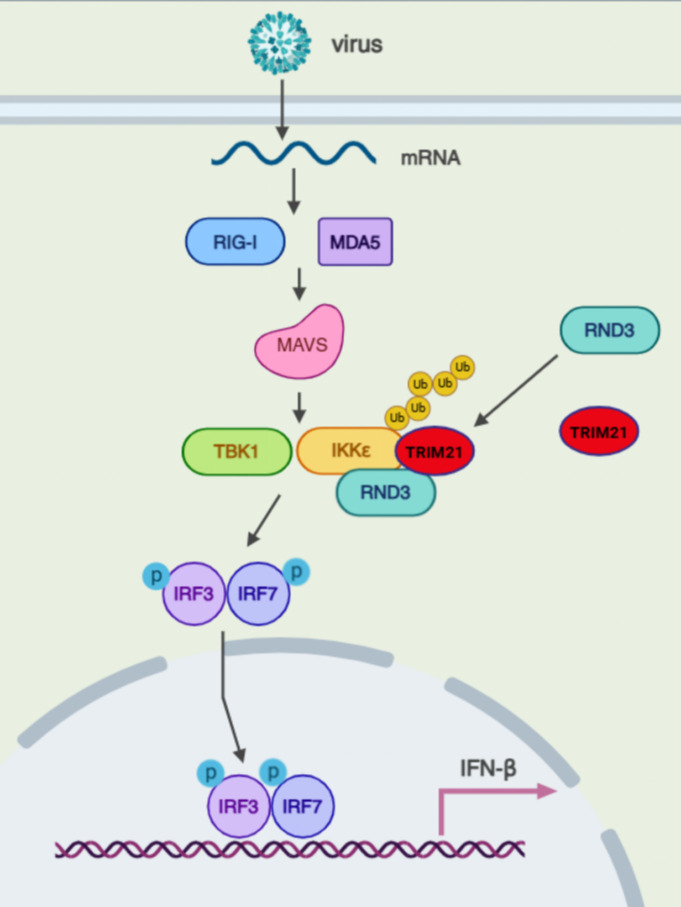
A working model for the role of RND3 proteins in regulating interferon-mediated signaling to mediate anti-EMCV replication.

The successful propagation of viruses depends on antagonizing the cellular mechanisms of the host while evading the innate immune response (20). Hosts have developed innate cellular defenses against infection by and replication of viruses. Importantly, RLR-mediated innate immune signaling pathways play important roles in recognizing viral RNA in the cytoplasm (21). Moreover, the activation of RLR signaling generates an antiviral response that is required for the host suppression of viral replication (22). In this study, a screen identified RND3 as a potential viral restriction factor in hosts. Moreover, RND3 inhibited EMCV and VSV replication in a dose-dependent manner. Mechanistically, RND3 enhanced IKKε protein stability by interacting with IKKε and, in this way, promoted virus-triggered IFN-β expression. Furthermore, we demonstrated that in cells, virus infection significantly downregulated RND3 protein levels, promoting host antiviral response escape (Fig. 9).

An increasing number of host-restricted factors that target positive regulatory functions of the antiviral immune system are being identified. For example, IFI44 restricts the replication of respiratory syncytial virus (23); ZFP36 significantly suppresses human coronavirus OC43 and FMDV replication (24, 25); ZDHHC1 positively regulates MITA/STING-dependent innate immune signaling to protect against DNA viruses (26); NEURL3 promotes IFN-β-mediated antiviral effects by catalyzing the K63-linked ubiquitination of IRF7 (27); and RND3 inhibits Chikungunya virus (CHIKV) replication (18). In this study, we screened IFI44, ZFP36, ZDHHC1, RND3, and NEURL3 for anti-EMCV replication effects *in vitro*. The results showed that RND3 more significantly inhibited EMCV replication than the other genes did.

RND3 belongs to the Rho GTPase family, and Rho GTPases are involved in PRR-mediated immune responses. In monocytes, Rac1 and RhoA play crucial roles in TLR2-induced NF-κB activation through the Rac1/PI3K/protein kinase B pathway and, at least in part, through atypical protein kinase C (28, 29). Rac1 is also involved in the TLR1-mediated signaling pathway in epithelial cells (30). The TLR7/9-mediated production of type I IFN requires the involvement of Rac in DCs (31). Therefore, we explored whether RND3 regulated PRR-mediated immune responses *in vitro*. We found that RND3 promoted the SeV- and poly(I:C)-mediated activation of the RLR signaling pathway in a dose-dependent manner. Herein, we demonstrated that RND3 acted as a positive regulator of IκB kinase ε (IKK-ε) to enhance the IFN-I signaling response. We found that RND3 promoted the expression of the adaptor proteins MAD5/RIG-I/MAVS/TBK1/IKKε to mediate IFN-β promoter activity. Additionally, RND3 specifically interacted with IKKε. In the RLR signaling pathway, IKKε plays an essential role in the activation of interferon production. IKKε and TBK1 are members of the IKK family and phosphorylate the transcription factors IRF3 and IRF7, which are required for type I IFN production. To resist viral replication, host proteins target IKKε to activate the antiviral immune response. For example, DLG1 suppresses negative-sense RNA virus replication by inhibiting the autophagic degradation of IKKε (32). Herein, we found that RND3 interacted with IKKε and enhanced protein stability but not the expression of RIG-I/MAVS/TBK1.

Protein ubiquitination is an important post-translational covalent modification process that has been extensively demonstrated to regulate signaling pathways in immune regulation (33). The ubiquitination modification of adaptor proteins occurs, which mediates the activation of RLR signaling pathways and is also essential for the production of antiviral ISGs (34). The E3 ubiquitin ligase TRIM6 interacts with IKKε, catalyzing the synthesis of unanchored K48 linked polyubiquitin chains, thereby triggering the activation of IKKε (35). In our research, we found that RND3 potentiates IFN-β production through direct interaction with IKKε. Subsequent IP/MS screening identified TRIM21 as a key interactor of RND3. Further investigation into the underlying mechanism revealed that RND3 recruits TRIM21 to facilitate this process. Extensive research has established that TRIM21 exerts regulatory control over the RLR signaling cascade through its E3 ubiquitin ligase activity, specifically by facilitating ubiquitin conjugation to adaptor proteins within this antiviral pathway. TRIM21 has been shown to promote K48-linked ubiquitination and degradation of STING and MAVS, thereby suppressing the host antiviral immune responses (36). In addition, TRIM21 mediates the ubiquitination and proteasomal degradation of IRF7, promoting the replication of Rabies virus (37). TRIM21 enhances or impairs IRF3-mediated gene expression by preventing IRF3 ubiquitination and degradation upon RNA virus infection (38). In addition, RND3 can also catalyze protein degradation by the ubiquitin-proteasome pathway. For example, RND3 suppresses endothelial cell pyroptosis in atherosclerosis by interacting with TRAF6 and promoting its K48-linked ubiquitination, which in turn inhibits the activation of the NF-κB pathway (39). Significantly, our findings demonstrate that RND3-mediated TRIM21 recruitment induces K63-linked ubiquitination of IKKε, promoting its stabilization and signaling activation. Our data suggest that RND3 targets IKKε protein to promote IFN-β production by hosts via a novel antivirus mechanism.

As a negative feedback regulator, a key finding is the downregulation of RND3 induced by the virus. Notably, this occurs primarily at the transcriptional level, as consistently observed with two distinct EMCV strains. This conserved mechanism suggests that suppressing RND3 transcription is a fundamental immune evasion strategy for EMCV. While the precise viral protein responsible remains to be fully identified, its effect—transcriptional suppression—points toward a sophisticated viral interference with host gene regulation, the details of which are a compelling focus of our ongoing research.

In conclusion, our study reveals that the host protein RND3 is a crucial positive regulator of antiviral signaling, which is specifically targeted by EMCV. A key finding is that two distinct EMCV strains consistently downregulate RND3 at the transcriptional level, revealing a conserved immune evasion strategy. We propose that viral suppression of RND3 may be a critical determinant of susceptibility and pathogenesis, establishing a foundation for future translational research on this zoonotic virus.

## Data Availability

The nucleotide sequences analyzed in this study were obtained from the public NCBI RefSeq database. The specific reference sequences and their direct access links are as follows: Homo sapiens IFI44: NM_006417.5, Homo sapiens ZFP36: NM_003407.5, Homo sapiens ZDHHC1: NM_013304.3, Homo sapiens NEURL3: NM_001285485.2, Homo sapiens RND3: NM_001254738.1, and Mus musculus RND3: NM_028810.2.

## SUPPLEMENTAL MATERIAL

Fig. S1 spectrum.01745-25-s0001.docx RND3 activates type I interferon production in MEFs cells.

ASM does not own the copyrights to Supplemental Material that may be linked to, or accessed through, an article. The authors have granted ASM a non-exclusive, world-wide license to publish the Supplemental Material files. Please contact the corresponding author directly for reuse.
