# Supplementary material for: RND3 restricts encephalomyocarditis virus replication by promoting IKKε ubiquitination and type I interferon production
Source: Microbiol Spectr. 2025 Dec 15;14(2):e01745-25. doi: 10.1128/spectrum.01745-25 (PMC12889043; doi:10.1128/spectrum.01745-25)
Supplement: Fig. S1 — RND3 activates type I interferon production in MEFs cells. [file spectrum.01745-25-s0001.docx]

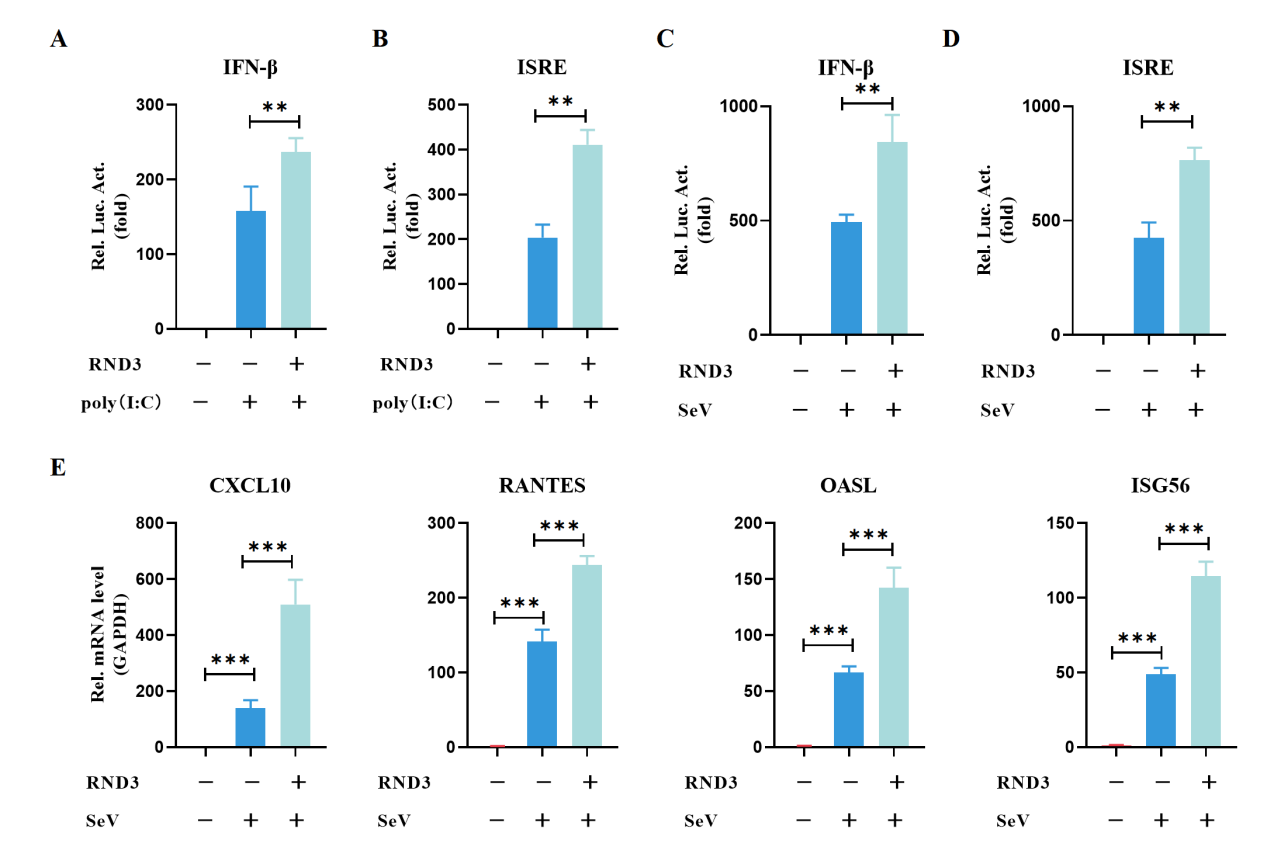


**Figure S1. RND3 activates type I interferon production in MEFs cells.** (A-B) MEFs cells were co‐transfected with pcDNA3.1‐Myc‐mRND3 (0.5 μg), pIFN-β‐luc (200 ng), pISRE‐luc (200 ng), and pRL‐TK (10 ng). After 24 h, the cells were stimulated with poly(I:C) for 12 h. Then, luminescence was detected using dual‐luciferase report. (C-D) MEFs cells were co‐transfected with pcDNA3.1‐Myc‐mRND3 (0.5 μg), pIFN-β‐luc (200 ng), pISRE‐luc (200 ng), and pRL‐TK (10 ng). After 24 h, the cells were stimulated with SeV at MOI=1 for 12 h. Then, luminescence was detected using dual‐luciferase report. (E) MEFs cells were transfected with pcDNA3.1‐Myc‐mRND3 (1 μg), or pcDNA3.1-Myc (1 μg). After 24 h, the cells were stimulated with SeV at MOI=1 for 12 h. Then, CXCL10, RANTES, OASL and ISG56 mRNA levels were detected using qRT-PCR. The results are presented as means ± standard deviations.**, *P* <0.01; ***, *P* <0.001 versus control.
